# The Southern Journal of Medicine and Pharmacy

**Published:** 1846-03

**Authors:** 


*The Southern Journal of Medicine and Pharmacy.* Edited by J. Lawrence Smith, M. D., and S. D. Linkler, M. D. Vol. 1, No. 1, for January, 1846, bi-monthly. Charleston, S. C.: Burges & James.

We have received the first number of the above named publication. It contains one hundred and twenty pages, is neatly printed and ably con ducted. The subscription price is four dollars per annum. We should suppose such a publication needed in Charleston, and we hope it may have an extensive circulation.

